# Comparative Evaluation of Mechanical and Physical Properties of Mycelium Composite Boards Made from *Lentinus sajor-caju* with Various Ratios of Corn Husk and Sawdust

**DOI:** 10.3390/jof10090634

**Published:** 2024-09-05

**Authors:** Praween Jinanukul, Jaturong Kumla, Worawoot Aiduang, Wandee Thamjaree, Rawiwan Oranratmanee, Umpiga Shummadtayar, Yuttana Tongtuam, Saisamorn Lumyong, Nakarin Suwannarach, Tanut Waroonkun

**Affiliations:** 1 Faculty of Architecture, Chiang Mai University, Chiang Mai 50200, Thailand; praween_ji@cmu.ac.th (P.J.); rawiwan.o@cmu.ac.th (R.O.); umpiga.sh@cmu.ac.th (U.S.); yuttana.t@cmu.ac.th (Y.T.); 2 Office of Research Administration, Chiang Mai University, Chiang Mai 50200, Thailand; jaturong_yai@hotmail.com (J.K.); worawoot.aiduang@cmu.ac.th (W.A.); 3 Department of Biology, Faculty of Science, Chiang Mai University, Chiang Mai 50200, Thailand; saisamorn.l@cmu.ac.th; 4 Center of Excellence in Microbial Diversity and Sustainable Utilization, Chiang Mai University, Chiang Mai 50200, Thailand; 5 Department of Physics and Materials Science, Faculty of Science, Chiang Mai University, Chiang Mai 50200, Thailand; wandee.th@cmu.ac.th; 6 Academy of Science, The Royal Society of Thailand, Bangkok 10300, Thailand

**Keywords:** biodegradable materials, lignocellulosic substrate, mycelium-based composites, mushroom mycelium, waste recycling

## Abstract

Mycelium-based composites (MBCs) exhibit varied properties as alternative biodegradable materials that can be used in various industries such as construction, furniture, household goods, and packaging. However, these properties are primarily influenced by the type of substrate used. This study aims to investigate the properties of MBCs produced from *Lentinus sajor-caju* strain CMU-NK0427 using different ratios of sawdust to corn husk in the development of mycelium composite boards (MCBs) with thicknesses of 8, 16, and 24 mm. The results indicate that variations in the ratios of corn husk to sawdust and thickness affected the mechanical and physical properties of the obtained MCBs. Reducing the corn husk content in the substrate increased the modulus of elasticity, density, and thermal conductivity, while increasing the corn husk content increased the bending strength, shrinkage, water absorption, and volumetric swelling. Additionally, an increase in thickness with the same substrate ratio only indicated an increase in density and shrinkage. MCBs have sound absorption properties ranging from 61 to 94% at a frequency of 1000 Hz. According to the correlation results, a reduction in corn husk content in the substrate has a significant positive effect on the reduction in bending strength, shrinkage, and water absorption in MCBs. However, a decrease in corn husk content shows a strong negative correlation with the increase in the modulus of elasticity, density, and thermal conductivity. The thickness of MCBs with the same substrate ratio only shows a significant negative correlation with the modulus of elasticity and bending strength. Compared to commercial boards, the mechanical (bending strength) and physical (density, thermal conductivity, and sound absorption) properties of MCBs made from a 100% corn husk ratio are most similar to those of softboards and acoustic boards. The results of this study can provide valuable information for the production of MCBs and will serve as a guide to enhance strategies for further improving their properties for commercial manufacturing, as well as fulfilling the long-term goal of eco-friendly recycling of lignocellulosic substrates.

## 1. Introduction

A current issue with synthetic materials, including cement, concrete, and polymers, is their environmental impact, particularly their contribution to pollution problems [1,2,3]. These materials are incapable of deterioration and take hundreds of years to degrade, causing them to accumulate in landfills, oceans, and the natural environment [4,5]. Furthermore, the production of synthetic materials frequently requires complex equipment, high costs for raw materials, processing, and product development, as well as high energy consumption during production [6,7,8]. Consequently, a worldwide campaign has been established to create and use more sustainable materials and technologies [7,9,10]. In recent years, there has been a growing interest in lignocellulose-based composites due to their renewable and biodegradable materials, which often renders them more environmentally friendly compared to synthetic materials [11,12]. However, the widespread use of formaldehyde-based resins as adhesives for bonding lignocellulose-based composites limits the advancement of fully natural composite materials. Furthermore, formaldehyde emissions from these products are classified as carcinogenic and harmful to human health [13,14,15]. In response to these concerns, researchers have shifted their focus towards developing natural, ecologically sustainable alternatives to replace toxic chemicals used in lignocellulose-based materials [16,17].

One of the promising alternatives is mycelium, derived from fungi, which shows great potential as a green adhesive material, replacing synthetic adhesives in lignocellulose-based composite production and creating mycelium-based composites (MBCs) [18,19,20,21,22,23,24]. MBCs represent the biological processes during the vegetative growth phase of fungal mycelium, characterized by a complex network with the unique ability to digest and adhere to organic substrate surfaces under ambient conditions, acting as a natural self-assembling adhesive [25]. Beyond its adhesive properties, mycelium exhibits structural binding characteristics by generating interconnecting fibrous threads consisting of chitin- and beta-glucan-based structural oligosaccharides. These oligosaccharides contribute to forming a robust and cohesive network within the mycelial structure [26]. This transformative process of MBCs converts lignocellulose waste into valuable resources, offering an eco-friendly alternative material [6,27,28]. Moreover, the process of MBC production is characterized by low energy consumption, low processing costs, a low carbon footprint, biodegradability, and an attractive range of properties for construction materials, board products, packaging, and foam-like materials, contributing to an overall reduction in waste and pollution [21,23,24,29,30,31]. Based on the fungal mycelial network, the monomitic hyphal system consists solely of generative hyphae. While the dimitic hyphal system forms both generative and skeletal hyphae and the trimitic hyphal system comprises three types of hyphae (generative, skeletal, and binding hyphae) [32]. MBCs made from dimitic and trimitic hyphae have higher qualities than those formed from monomitic hyphae because the skeletal and binding hyphae in these types have thicker walls, higher densities, and greater hardness [22]. Various lignocellulosic materials, particularly from the agricultural sector, including cotton, straw, sawdust, woodchips, and rice husk, have been utilized as organic substrates in the production of MBCs [6,22,23,24,25,28]. Additionally, lignocellulosic materials have been selected and used in the production of MBCs based on their availability in each country. Remarkably, the characteristics of MBCs can be directly influenced by different fungal mycelial network and substrate types [8,14,21,24,27,28,29]. In our previous study, *Lentinus sajor-caju* (dimitic hyphal system) was found to have a great deal of potential for developing MBCs from sawdust and corn husk [33]. However, the different ratios of sawdust to corn husk in the production process are still necessary for the development of MBCs. To date, there are no reports on MBCs made from varying ratios of corn husks and sawdust. Therefore, the purpose of this study was to investigate the mechanical (modulus of elasticity and bending strength) and physical properties (density, shrinkage, water absorption, volumetric swelling, thermal conductivity, and sound absorption) of mycelium composite boards (MCBs) produced from *L. sajor-caju* using different ratios of sawdust to corn husk. The information obtained from this study can be valuable for developing MBCs as a type of bio-board with properties suitable for environmentally friendly architecture.

## 2. Materials and Methods

### 2.1. Fungal Strain

The pure culture of *L. sajor-caju* strain CMU-NK0427 was obtained from the Culture Collection of the Center of Excellence in Microbial Diversity and Sustainable Utilization, Faculty of Science, Chiang Mai University, Thailand, and used in this study. This fungus was cultured on potato dextrose agar (PDA, Conda, Madrid, Spain) and incubated at 30 °C.

### 2.2. Sources of Substrate and Preparation

Two different types of wood and agricultural residues, namely rubber tree sawdust and corn husk, were used as substrates for the present study. These residues were from a sawmill and agricultural regions situated in Chiang Mai Province, Thailand. Both substrates were dried at 60 °C in an oven for 72 h. Subsequently, each substrate was then ground in a woodchipper and sieved. Particles ranging in size from 5 to 20 mm were collected and used.

### 2.3. Preparation of Mycelial Inoculum

The mycelial inoculum was prepared using sorghum seeds as a nutrient source. Initially, sorghum seeds were boiled for 20 min and then cooled. Subsequently, 100 g of boiled grains was placed in a glass bottle with a cotton swab inserted. The bottle was autoclaved at 121 °C for 20 min and then gradually cooled at room temperature over 24 h. Following this process, mycelial plugs (1 × 1 cm) obtained from pure culture of *L. sajor-caju* on PDA were transferred to a 325 mL glass bottle (5 plugs per bottle). After that, the inoculated bottle was incubated for two weeks at 30 °C to allow the sorghum seeds to be completely covered with mycelium for use as an inoculum.

### 2.4. Preparation of MBCs

The preparation of MBCs in this study followed the methods of Aiduang et al. [33]. Five different ratios based on the dry weight of corn husk and sawdust were designed in this study, including 100% corn husk, 75% corn husk with 25% sawdust, 50% corn husk with 50% sawdust, 25% corn husk with 75% sawdust, and 100% sawdust. Each mixed substrate was added with 5% rice bran, 1% calcium carbonate, 2% calcium sulfate, and 0.2% sodium sulfate. The moisture content of each mixed substrate was adjusted at 60% relative humidity by adding water. Then, 500 g of each mixed substrate was carefully placed in a polypropylene bag with dimensions of 3.5 inches in width and 12.5 inches in length. The bag was securely sealed. These sealed bags were then inserted into polyvinyl chloride rings and covered with paper before being subjected to autoclaving at 121 °C for 60 min. After autoclaving, a cooling period of 24 h at room temperature was allowed. Five grams of mycelial inoculum was inoculated onto the top of the substrate of each bag. The inoculated bags were incubated at 30 °C and fungal mycelia were observed to completely cover the substrate after 35 days of incubation.

### 2.5. Preparation of MCB Molds

The molds used in MCB production were meticulously fabricated from acrylic sheets with dimensions of 410 mm × 570 mm, featuring a side margin of 20 mm, resulting in MCB specimens with sizes of 370 mm × 530 mm. The MCBs were then cut to standard sizes for testing material properties. It is important to note that the molds have varying thicknesses, mirroring the diversity observed in current board materials. For this study, three representative thicknesses of 8 mm, 16 mm, and 24 mm were intentionally selected to assess the influence of mycelium board thicknesses on its properties.

### 2.6. MCB Production

**Figure 1:**
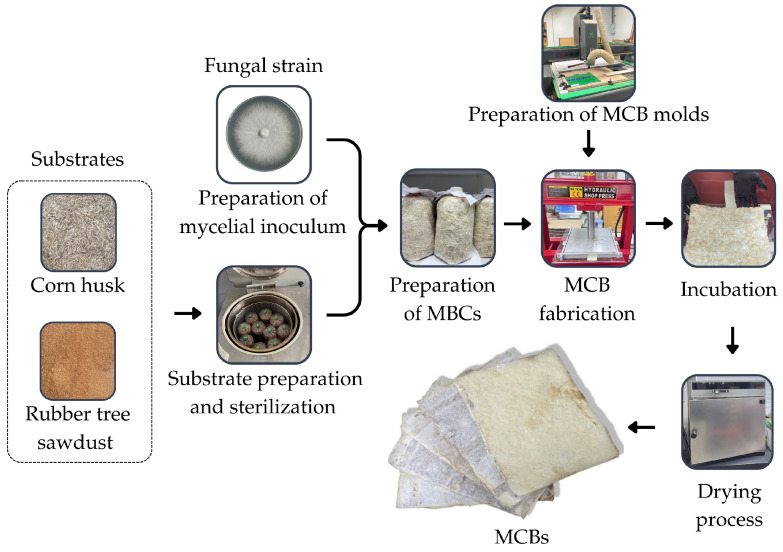
Scheme for the MCB production process in this study.

The substrate covered by fungal mycelia inside the bags were carefully transferred to designated molds corresponding to each thickness. A total of 800, 1600, and 2400 g were used for 8, 16, and 24 mm thick molds, respectively. Then, cold pressing was conducted using a press (Shop press ZX0901 E–1, New Taipei, Taiwan). The pressing process involved exerting a controlled force of 2 MPa for a precise duration of 10 min. Following cold pressing, the molds underwent incubation at 30 °C for a period of three weeks to allow further development of the samples. Then, specimens were removed and incubated for another 7 days. Subsequently, the samples were carefully transferred to an oven at 70 °C to dry completely for 72 h. The scheme for the MCB production process in this study is shown in Figure 1.

### 2.7. Specimen Preparation for Determination of Mechanical and Physical Properties

**Figure 2:**
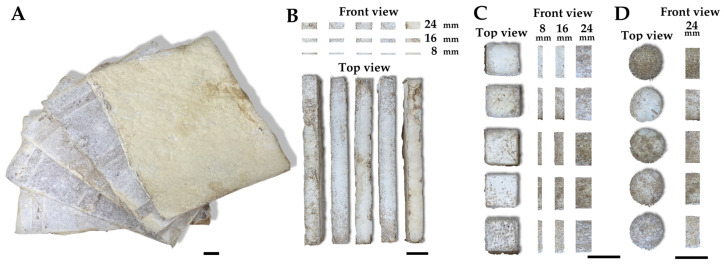
Example specimens of MBCs obtained from *L. sajor-caju* and corn husk and sawdust in this study. Mycelium composite boards (**A**). Specimens for modulus of elasticity and bending strength testing (**B**), specimens for water absorption measurement, thickness of swelling, shrinkage, and thermal conductivity measurement (**C**), and specimens for sound absorption coefficient measurement (**D**). Scale bar = 50 mm.

Each MBC, with varying substrate ratios and thicknesses (Figure 2A), was cut into specimens for testing mechanical and physical properties (Table 1). Three specimens were used for testing each property. Specimens for testing the modulus of elasticity (MOE) and bending strength (BS) were cut to a width of 50 mm and a length of 530 mm, as shown in Figure 2B. Specimens for testing density, water absorption, swelling thickness, and thermal conductivity were cut to a width and length of 50 mm, as shown in Figure 2C. Additionally, specimens for measuring the sound absorption coefficient were cut to a diameter of 50 mm, as shown in Figure 2D. Note that specimens for measuring thermal conductivity and sound absorption coefficient were cut from an MCB with a thickness of 24 mm due to limitations with the testing equipment. The material properties were examined at the Science and Technology Service Center and the Department of Physics and Materials Science, Faculty of Science, Chiang Mai University, Chiang Mai, Thailand.

### 2.8. Determination of Mechanical Properties

#### 2.8.1. Modulus of Elasticity

The testing procedure was conducted utilizing a universal testing machine employing the centralized concentration loading method, as outlined in BS EN 310:1993 [34,35]. The modulus of elasticity (MOE) was determined using the formula [l_1_^3^ (F_2_ − F_1_)]/4 bt^3^ (a_2_ − a_1_), where (F_2_ − F_1_) represents the increased load applied along the linear segment of the load–deflection curve, measured in newtons (N). ‘F_1_’ corresponds to approximately 10% of the maximum load, while ‘F_2_’ corresponds to approximately 40% of the maximum load. The width of the specimen (‘b’) and its thickness (‘t’) are measured in millimeters (mm), and ‘a_2_ − a_1_’ denotes the deflection of the specimen at mid-span. The MOE is expressed in units of MPa.

#### 2.8.2. Bending Strength

The experiments were conducted using a universal testing machine based on the centralized concentration loading method, enabling the evaluation of bending strength (BS) [36,37]. The formula for BS is derived from (3F_max_l_1_)/(2 bt^2^) according to BS EN 310:1993, where ‘F_max_’ denotes the maximum load measured in newtons (N), ‘l_1_’ refers to the distance between the centers of the supports measured in millimeters (mm), ‘b’ represents the width of the specimen in millimeters (mm), and ‘t’ indicates the thickness of the specimen. The BS test yields resulted in units of MPa.

### 2.9. Determination of Physical Properties

#### 2.9.1. Density

Density tests were conducted on the MBCs to determine the mean density associated with each specific board type. The testing procedure strictly adhered to the guidelines stipulated in the British code of standards, specifically BS EN 323 [36,38]. The equation of density (kg/m^3^) = M/V, where W = the mass of the specimen (kg) and V = the volume of the specimen (m^3^).

#### 2.9.2. Shrinkage

The shrinkage of each specimen was assessed and computed using wet and dry volumes, following the procedure outlined by Elsacker et al. [20]. This reduction was quantified as a shrinkage percentage (%), calculated as follows: Shrinkage (%) = [(V_1_ − V_2_)/V_1_] × 100, where V_1_ represents the wet volume of the specimen (m^3^) and V_2_ is the dry volume of the specimen (m^3^).

#### 2.9.3. Water Absorption

Water absorption tests followed the ASTM D1037:2002 standard [37,39]. The specimens were dried at 70 °C until achieving a stable mass. Following this, the initial mass of each specimen was precisely measured to ensure data accuracy. After measurement, the specimens were submerged in deionized water for a total duration of 84 h, with weight measurements taken at precise time intervals of 2, 4, 6, 12, 18, 24, 36, 48, 60, 72, and 84 h. Water absorption (%) was calculated using the formula (W − D) × 100)/D, where W represents the wet mass (kg) and D represents the dry mass (kg) [40].

#### 2.9.4. Volumetric Swelling

The determination of volumetric swelling of MCB specimens followed the protocols of the ASTM D1037:2002 standard [37]. Initial sizing (width × length × thickness) measurements (T_I_) were accurately recorded using a vernier caliper. Subsequently, the samples underwent immersion in water for designated durations of 84 h, respectively. Post-immersion, the final sizing (T_f_) of each sample was measured and documented. The percentage of size change by each sample was calculated using the equation for volumetric swelling: (T_f_ − T_i_) × 100/T_i_, where T_f_ represents the final volume (m^3^) and T_i_ denotes the initial volume (m^3^) [41].

#### 2.9.5. Thermal Conductivity

Thermal conductivity in this study was conducted following ISO 22007-2:2022 [41,42]. The measurement was conducted at room temperature employing the hot wire technique utilizing the Transient Hot Bridge apparatus (LINSEIS, THB-1). Two specimens were measured, with a sensor probe positioned between their surfaces in a sandwich setup configuration [43].

#### 2.9.6. Sound Absorption Coefficient

The sound absorption coefficient in this study was quantified following the established protocols delineated in ISO 10534-2 [44], employing a Kundt’s tube apparatus [45,46]. Measurements were conducted at discrete frequencies of 250, 500, and 1000 Hz, and outcomes were represented as a percentage.

### 2.10. Statistical Analysis

The data obtained from each experiment were analyzed through one-way analysis of variance (ANOVA) using the SPSS program, v22, for Windows. Subsequently, Duncan’s multiple range test was utilized to ascertain significant differences (*p* ≤ 0.05) among the mean values. The Pearson correlation coefficients (*r*) for the ratio of substrate and thickness with the properties of MCBs were analyzed using the SPSS program at a significance level of *p* < 0.05.

## 3. Results and Discussion

### 3.1. Mechanical Properties of MCBs

#### 3.1.1. Modulus of Elasticity

**Figure 3:**
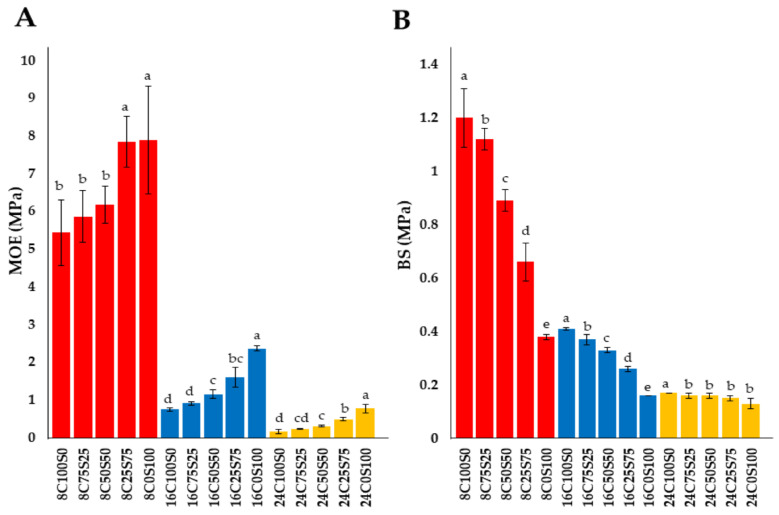
Modulus of elasticity [MOE; (**A**)] and bending strength [BS; (**B**)] of MCBs in this study. Red, blue, and yellow bars indicate MCBs with thicknesses of 8 mm, 16 mm, and 24 mm, respectively. The different letters within the same group of MCB thickness indicate a significant difference according to Duncan’s multiple range test (*p* ≤ 0.05).

The MOE values obtained in 8 mm, 16 mm, and 24 mm thick specimens ranged from 5.44 to 7.88 MPa, 0.75 to 2.36 MPa, and 0.17 to 0.77 MPa, respectively (Figure 3A). According to our results, the MOE values ranged from 0.17 to 7.88 MPa and fell within the previously documented ranges of MOE values observed in MBCs (0.14 to 97.00 MPa) [20,22,31,46]. The results showed that the different ratios of corn husks to sawdust affected the MOE value. An increase in the ratio of sawdust led to an increase in the MOE value for each thickness. These outcomes were consistent with previous studies showing that the type of lignocellulosic residues and the ratio mixture used in the bio-fabrication of MBCs affect their modulus of elasticity [20,22,31,46]. Results showed that there was no significant difference in the MOE values of 8C25S75 and 8C0S100 specimens. Furthermore, the MOE value decreased as MBC thickness increased (Figure 3A). The maximum MOE value for the same substrate ratio was found at a thickness of 8 mm, followed by 16 mm and 24 mm. This result was supported by previous studies that found the elastic modulus of the material increases with decreasing thickness [47,48].

Kuznetsova et al. [49] found that an increase in the thickness of mycelial films made from *Ganoderma lucidum* and *Pleurotus eryngii* was associated with decreased MOE values. Additionally, Appels et al. [22] explained that thinner MBCs have a higher distribution and density of mycelial networks per unit volume compared to thicker MBCs, which enhances the material’s stiffness and resistance to deformation, contributing to a higher elastic modulus. According to the Pearson correlation (*p* < 0.05), the MOE value showed a significant strong negative correlation with a decreasing in the ratio of corn husk at the same thickness (*r* = −0.937 to −0.772, *p* < 0.001) (Table 2). This indicates that lower corn husk content is associated with a higher MOE value. The MOE value also showed a significant negative correlation with an increase in thickness at the same substrate ratio (*r* = −0.929 to −0.898, *p* < 0.001) (Table 3). Appels et al. [22] also found that the hot-pressing process increased the MOE value compared to the cold-pressing process.

#### 3.1.2. Bending Strength

The BS values of specimens obtained in this study are shown in Figure 3B. The BS values were 0.38 to 1.26 MPa, 0.16 to 0.41 MPa, and 0.13 to 0.17 MPa for specimens with thicknesses of 8 mm, 16 mm, and 24 mm, respectively. When compared to previous studies, the BS values from this study (0.13 to 1.26 MPa) fall within the reported range of 0.07 to 4.40 MPa [6,22,50,51,52]. The results revealed that the different ratios of corn husks to sawdust affected the BS value, and an increase in the ratio of sawdust led to a decrease in the BS value for each thickness. The highest BS values of the obtained MCB in this study was discovered in the MCB made from 100% corn husk in each thickness. In contrast, the 100% sawdust MCBs had the lowest bending strength. Similarly, numerous previous studies have demonstrated that the flexural strength of MBCs was influenced by the type of substrate, type of mycelia network, and pressing method [22,53]. Generally, bending strength is influenced by the proportion of lignin and cellulose in the substrate. The proportion of lignin and cellulose in rubber tree sawdust ranges from 0.86 to 1.35, which is higher than in corn husks, which ranges from 0.22 to 0.33 [54,55,56,57]. Previous studies have found that substrates with a higher ratio of lignin and cellulose exhibit lower bending strength [58,59]. Similarly, this study found that MCBs made from substrates with increasing sawdust content showed a higher ratio of lignin to cellulose, which resulted in reduced bending strength. Furthermore, *L. sajor-caju* is a white-rot fungus that primarily breaks down lignin in its growing substrate [60]. This may enhance lignin degradation and lead to a higher cellulose content in the substrate, which is also associated with increased bending strength. In this study, the bending strength (BS) value showed a significant positive correlation with a decrease in the ratio of corn husks at the same thickness (*r* = 0.764 to 0.977, *p* < 0.001) (Table 2), indicating that lower corn husk content is associated with a lower BS value. A strong negative correlation between the BS value and an increase in thickness at the same substrate ratio (*r* = −0.957 to −0.919, *p* < 0.001) was observed, according to Pearson correlation analysis (*p* < 0.05) (Table 3).

### 3.2. Physical Properties of MCBs

#### 3.2.1. Density

**Figure 4:**
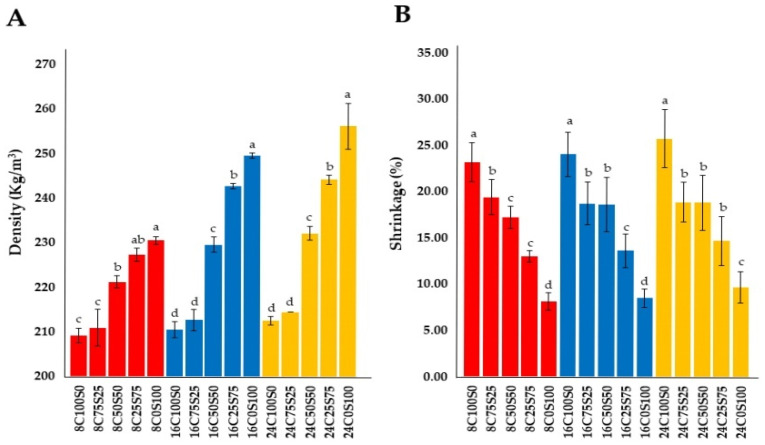
Density (**A**) and shrinkage (**B**) of MCBs in this study. Red, blue, and yellow bars indicate MCBs with thicknesses of 8 mm, 16 mm, and 24 mm, respectively. The different letters within the same group of MCB thickness indicate a significant difference according to Duncan’s multiple range test (*p* ≤ 0.05).

The density of the MCBs in this study is displayed in Figure 4A. The results exhibited that the obtained density value varied according to the ratio of substrates used. The highest density was found in the MCB made from 100% sawdust in each thickness. The findings indicated that the decrease in MCB density varied with the increase in the amount of corn husk added. Density values in this investigation ranged from 209.17 to 256.28 kg/m^3^, and were in the range of 25 to 954 kg/m^3^ from previous reports [15,22,57,61,62,63,64,65]. In addition, the density at the same substrate ratio increased with increasing thickness. The results are consistent with several previous studies, which reported that substrate type and ratio, substrate particles, volume fraction, and pressing process have a major impact on the density of MBCs [62,66,67].

According to the Pearson correlation (*p* < 0.05), the density showed a significant strong negative correlation with a decrease in the ratio of corn husks at the same thickness (*r* = −0.975 to −0.635, *p* < 0.001). This indicates that a lower corn husk content has a higher density value. It also showed a significant positive correlation with an increase in thickness at substrate ratios of 50% corn husks and 50% sawdust, 25% corn husk and 75% sawdust, and 100% sawdust (*r* = 0.898 to 0.929, *p* < 0.001) (Table 2). However, the substrate ratios of 100% corn husk (*r* = −0.635, *p* = 0.006) and 75% corn husk and 25% sawdust (*r* = −0.555, *p* = 0.120) showed a non-significant correlation with an increase in thickness (Table 3).

#### 3.2.2. Shrinkage

Shrinkage value is an important aspect of the physical properties of MCBs, primarily resulting from the dehydration process during drying and depending on the substrate type [20,53,62,64]. According to this study, different ratios of corn husks to sawdust affected the shrinkage value, as shown in Figure 4B. MCBs produced from 100% corn husk exhibited the highest shrinkage values at each thickness, while the lowest values were observed in MCBs made from 100% sawdust. An increase in the ratio of sawdust led to a decrease in shrinkage values for each thickness. Additionally, the obtained shrinkage values ranged between 8.15% and 25.78%. Prior to this study, the shrinkage values of MBCs were reported to be in the range of 2.78% to 17% [12,20,62,65,66,67,68,69]. Remarkably, the shrinkage values obtained from ratios of 100% corn husk (23.20% to 25.78%), 75% corn husk with 25% sawdust (18.89% to 19.39%), and 50% corn husk with 50% sawdust (17.19% to 18.86%) at 8 mm, 16 mm, and 24 mm thicknesses were higher than previously reported values. Therefore, it is crucial to select a suitable substrate in order to control the shrinkage value of MBCs. According to the Pearson correlation (*p* < 0.05), a decrease in the ratio of corn husks at the same thickness showed a significantly strong positive correlation with the shrinkage value (*r* = 0.968 to 0.992, *p* < 0.001 to 0.007) (Table 2). This indicates that lower corn husk content is associated with a lower shrinkage value. However, the shrinkage value did not show a significant positive correlation with thickness at the same substrate ratio (*p* < 0.05) (Table 3).

#### 3.2.3. Water Absorption

**Figure 5:**
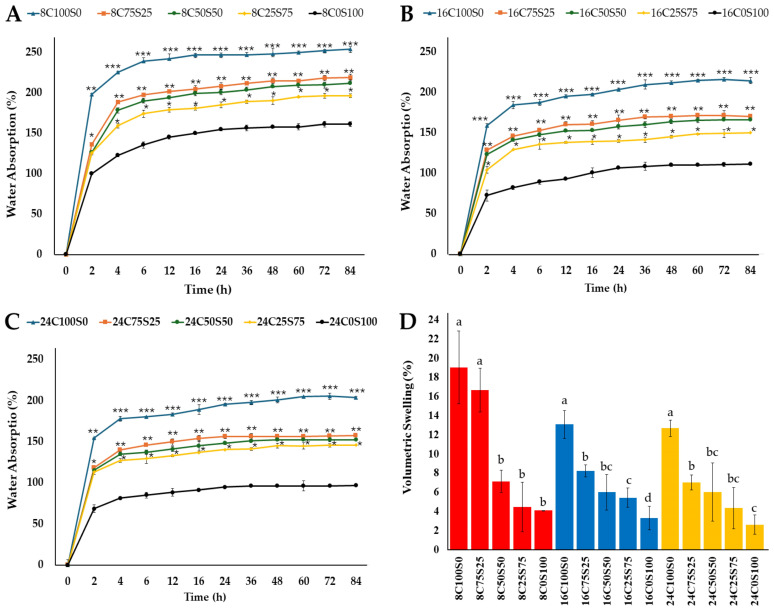
Water absorption behavior of MBC specimen’s thickness at 8 mm (**A**), 16 mm (**B**), and 24 mm (**C**) and volumetric swelling (**D**) in this study. *, **, *** in subfigures (**A**–**C**) indicate significant differences according to Duncan’s multiple range test (*p* ≤ 0.05). Red, blue, and yellow bars in subfigures (**D**) indicate MCBs with thicknesses of 8 mm, 16 mm, and 24 mm, respectively. The different letters within the same group of MCB thickness in subfigures (**D**) indicate a significant difference according to Duncan’s multiple range test (*p* ≤ 0.05).

The water absorption ability of the MCBs, obtained from different ratios of corn husks and sawdust and varying thicknesses, was assessed by immersing the MCB specimens in water for 84 h. The percentages of water absorption values over the period from 0 to 84 h are presented in Figure 5A–C. It was found that the water absorption ability of the MCBs made from 100% corn husks, in all thicknesses, increased over a 16 h period and slowly stabilized after 24 h. In contrast, the water absorption ability of the MCBs made from 100% sawdust increased over a period of 36 h and slowly stabilized after 48 h. Additionally, the water absorption ability of the mixture of corn husks and sawdust increased mostly over 24 h and slowly stabilized after 48 h. After 4 h, it was found that the water absorption for specimens with a 100% corn husk ratio showed the highest value, followed by specimens with a 75% corn husk and 25% sawdust ratio, a 50% corn husk and 50% sawdust ratio, and a 100% sawdust ratio. The water absorption for specimens with a 75% corn husk and 25% sawdust ratio and a 50% corn husk and 50% sawdust ratio was not significantly different over a period of 2 to 84 h. In addition, the increase in sawdust in MCBs decreased the water absorption. After 84 h, it was observed that MCBs produced from 100% corn husks displayed water absorption rates ranging between 204.01 and 256.11%, while MBCs produced from 100% sawdust exhibited water absorption rates ranging from 96.86 to 161.68%. The percentage of water absorbed by MCBs made of various sawdust and corn husk combinations ranged from 146.32 to 219.01%. The water absorption ability of MCBs in this study was within the range of 24.45 to 560%, as reported in previous studies when submerged in water for 24 to 192 h [6,20,22,23,30,33,66]. According to the results of several studies, MBCs are classified as hydrophilic materials, and the substrate type and fungal species influence their water absorption ability [20,22,23,30,33,66].

Based on the morphology of MBCs, water absorption capacity was found to correlate with the porous structure of the composites. Corn husks exhibit higher porosity compared to rubber tree sawdust [32]. Consequently, the higher ratio of corn husks used in the MCBs in this study led to increased water absorption. The water absorption capacity of MBCs is typically linked to the proportions of lignin and cellulose. Several previous studies have reported that lignin is a hydrophobic component, while cellulose is hydrophilic. Thus, a high proportion of lignin and cellulose in the substrate can decrease water absorption [32,33,70]. According to a comparison of the lignin and cellulose content of the two materials employed in this study, rubber tree sawdust has a larger lignin and cellulose proportion (0.86 to 1.35) than corn husks (0.22 to 0.33) [53,54,55,71]. As a result, MCBs with larger corn husk content have higher water absorption. Furthermore, the enhanced lignin degradation by *L. sajor-caju* results in a higher cellulose content in the substrate, which is associated with increased water absorption capacity. In this study, a decrease in the ratio of corn husks at the same thickness showed a significantly strong positive correlation with the water absorption ability (*r* = 0.973 to 0.937, *p* = 0.005 to 0.019) according to the Pearson correlation analysis (*p* < 0.05) (Table 2). This indicates that a lower corn husk content is associated with lower water absorption values. However, water absorption did not show a significant negative correlation with an increase in thickness at the same substrate ratio (*r* = −0.955 to −0.896, *p* = 0.193 to 0.210) (Table 3).

#### 3.2.4. Volumetric Swelling

After soaking in water for 84 h, the volumetric swelling of the MCBs in this study varied from 2.63 to 19.03% (Figure 5D) and fell within the previously reported ranges of volumetric swelling observed in MBCs (0.28 to 21%) [62,63,64,65,66,67,68,69]. The volumetric swelling rate of MCBs made from 100% corn husk was higher for all thicknesses compared to that of other MCBs. Subsequently, a decrease in corn husk ratio content in MCBs led to a reduction in volumetric swelling rate. It was found that MCBs with high water absorption also have high volumetric swelling. The outcome is in accordance with previous studies that found a direct correlation between volumetric swelling and the water absorption capability of the mycelium-based material [7,20]. In Table 2, the volumetric swelling rate showed a non-significant positive correlation (*r* = 0.949 to 0.943, *p* = 0.140 to 0.160) with a decrease in the ratio of corn husk at the same thickness. The volumetric swelling also exhibited a non-significant negative correlation with an increase in thickness at the same substrate ratio (*r* = −0.999 to −0.850, *p* = 0.220 to 0.940), as shown in Table 3.

#### 3.2.5. Thermal Conductivity Values

Thermal conductivity is the capacity of a material to conduct or transfer heat. In this study, only the 24 mm thick specimens were used due to limitations of the testing equipment. According to our results, the thermal conductivity values ranged from 0.037 to 0.079 W/m∙K (Table 4). These obtained values were within the previously documented range of thermal conductivity values observed in MBCs, which is 0.029 to 0.124 W/m·K [20,24,72,73,74]. The results showed that variations in the ratio of corn husk to sawdust led to different thermal conductivity values. An increase in sawdust ratio in MCBs led to an increase in the thermal conductivity value. The correlation between thermal conductivity value and a reduction in corn husk content showed a significant negative effect (*r* = −0.921, *p* = 0.026) (Table 2). In this study, it was found that the thermal conductivity values of MCBs were related to density, with higher thermal conductivity values associated with higher density values. The results are consistent with several previous studies that reported that the type of substrate used in mycelium-based material production influences thermal conductivity values and is related to density [53]. Based on our previous study [32], the morphological characteristics of MBCs produced from *L. sajor-caju* and lignocellulosic residues (including corn husks, sawdust, and rice straw) include larger pores and a dense mycelial network. Therefore, the possible mechanisms influencing the thermal conductivity of MCBs include porosity and air pockets within the material acting as thermal insulators, as well as the potential enhancement of heat transfer by a dense mycelial network, as reported in previous studies [75,76].

#### 3.2.6. Sound Absorption Coefficient

Sound absorption is one of the most important factors in producing suitable materials with MBCs. MCBs typically feature a porous structure due to the growth of mycelium and the presence of fibrous materials [77]. The possible mechanisms of sound absorption by MCBs include trapping sound waves within their porous structure; converting sound energy into heat through friction and viscous effects of the fibrous materials; and reducing sound intensity through disruption and scattering of sound waves by the mycelial network [78,79,80,81]. In this study, only specimens with a thickness of 24 mm were employed due to testing equipment restrictions. The percentage of sound absorption of MCBs at frequencies of 250, 500, and 1000 Hz is shown in Table 5. It was found that the percentage of sound absorption of MCBs varied with different substrate ratios used. The highest percentage of sound absorption at frequencies of 250, 500, and 1000 Hz was found in MCBs made using a 75% corn husk and 25% sawdust ratio. It was found that the percentage of sound absorption of the mixed substrate showed higher values than individual substrates, consistent with previous findings [6,20]. Moreover, the sound absorption coefficient of the substrate is influenced by substrate size and the pressing method [82]. In addition, Barta et al. [83] found that MBCs made from *Ganoderma lucidum* and *Trametes versicolor* showed different sound absorption abilities, indicating that the fungal species affects this property. Pelletier et al. [26] found that MBCs made from varied substrates (including residues of flax shive, hemp, kenaf, rice straw, sorghum stalk, and switch glass) showed 70–75% sound absorption at 1000 Hz. Interestingly, MCB specimens in this study made from pure corn husk and a mixture of corn husk with sawdust showed 84–94% sound absorption at 1000 Hz. According to the Pearson correlation (*p* < 0.05), a decrease in the ratio of corn husk content in MCBs showed a non-significant positive correlation with sound absorption at frequencies of 250 (*r* = 0.281, *p* = 0.647), 500 (*r* = 0.228, *p* = 0.712), and 1000 Hz (*r* = 0.740, *p* = 0.153) (Table 2). Our results suggest that the selection of substrate combination and sound frequencies should be taken into consideration when developing suitable MCBs for sound absorption materials. However, reports on the sound absorption coefficient of MBCs are limited; therefore, further studies are required.

### 3.3. Comparison of Properties with Commercial Boards

The properties of MCBs obtained in this study were compared to previous reports of MBCs and other commercial boards, as shown in Table 6. Based on mechanical properties, the BS values of MCBs were within the range of softboards and acoustic boards. However, the MOE values were lower than those of other conventional boards. Based on physical properties, the density values of some MCBs, including 8C0S100, 16C25S75, 16C0S100, 24C50S50, 24C25S75, and 24C0S100 in this study, fall within the density range of softboards. However, the shrinkage, water absorption, and volumetric swelling of MCBs were higher than those of other conventional boards. It was found that the thermal conductivity properties of MCBs were within the range of softboards and insulated boards. In addition, MCBs made from 100% corn husk exhibited thermal conductivity within the range of acoustic boards. According to the sound absorption coefficient, MCBs were in the range of acoustic boards, insulated boards, and softboards, with higher values than medium-density fiberboards, gypsum boards, and fiber cement boards.

## 4. Conclusions

This study investigated various mechanical and physical properties of MCBs derived from *L. sajor-caju*. The results showed that mechanical and physical properties of MCBs varied in substrate ratio and thickness. The different ratios of corn husk and sawdust showed a significant correlation with the MOE, BS, density, shrinkage, water absorption, and thermal conductivity of MCBs. The thickness of MCBs showed a significant correlation with MOE, BS, and density (with corn husk at less than 50%). It was found that the MOE (0.17–7.88 MPa), BS (0.13–1.26 MPa), density (209.17–256.08 kg/m^3^), water absorption (96.86–256.11%), volumetric swelling (2.63–19.03%), thermal conductivity (0.037–0.079 W/m·K), and sound absorption (61–94% at 1000 Hz) of MCBs were within the ranges reported in several previous studies. However, MCBs showed a higher shrinkage value (8.15–25.78%) compared to previous reports. Based on the mechanical (BS) and physical properties (density, thermal conductivity, and sound absorption), MCBs made from 100% corn husk are most similar to softboard and acoustic boards. Further improvement of the MOE, shrinkage, water absorption, and volumetric swelling of this MCB is still required. Improvement in the qualities of mycelium-based materials will be achieved through continued research and biotechnological developments, which will address the challenges of standardization, production costs, and commercial acceptability.

## Figures and Tables

**Table 1:** Information of MCBs created in this study.

Thickness (mm)	Ratio of Substrate Based on Dry Weight (%)		Specimen Name
	Corn Husk	Sawdust	
8	100	0	8C100S0
	75	25	8C75S25
	50	50	8C50S50
	25	75	8C25S75
	0	100	8C0S100
16	100	0	16C100S0
	75	25	16C75S25
	50	50	16C50S50
	25	75	16C25S75
	0	100	16C0S100
24	100	0	24C100S0
	75	25	24C75S25
	50	50	24C50S50
	25	75	24C25S75
	0	100	24C0S100

**Table 2:** Pearson correlation coefficients between the properties and a decreasing in the ratio of corn husk of MCBs with the same thickness.

Properties	Pearson Correlation Coefficients (*r*/*p*-Value)		
	8 mm	16 mm	24 mm
MOE	−0.772 */<0.001	−0.937 */<0.001	−0.923 */<0.001
BS	0.977 */<0.001	0.967 */<0.001	0.764 */<0.001
DS	−0.635 */<0.001	−0.975 */<0.001	−0.975 */<0.001
SK	0.992 */<0.001	0.973 */<0.001	0.968 */0.007
WA	0.973 */0.005	0.963 */0.009	0.937 */0.019
VS	0.943/0.160	0.949/0.140	0.944/0.160
TC	ND	ND	−0.921 */0.026
SAC (250 Hz)	ND	ND	0.281/0.647
SAC (500 Hz)	ND	ND	0.228/0.712
SAC (1000 Hz)	ND	ND	0.740/0.153

“*” indicates a significant correlation at a significance level of *p* < 0.05. *r* = Pearson correlation coefficients, MOE = modulus of elasticity, BS = bending strength, DS = density, SK = shrinkage, WA = water absorption, VS = volume swelling, SAC = sound absorption coefficient, and “ND” = not determined.

**Table 3:** Pearson correlation coefficients between the properties and an increase in thickness of MCBs with different substrate ratios.

Properties	Pearson Correlation Coefficients (*r*/*p*-Value)				
	C100S0	C75S25	C50S50	C25S75	C0S100
MOE	−0.898 */<0.001	−0.908 */<0.001	−0.921 */<0.001	−0.922 */<0.001	−0.929 */<0.001
BS	−0.945 */<0.001	−0.951 */<0.001	−0.957 */<0.001	−0.937 */<0.001	−0.919 */<0.001
DS	0.635/0.066	0.555/0.120	0.929 */<0.001	0.898 */<0.001	0.949 */<0.001
SK	0.980/0.125	0.752/0.549	0.921/0.256	0.990/0.089	0.958/0.186
WA	−0.946/0.200	−0.946/0.210	−0.955/0.193	−0.896/0.293	−0.951/0.200
VS	−0.894/0.297	−0.917/0.262	−0.850/0.353	−0.093/0.940	−0.999/0.220

“*” indicates a significant correlation at a significance level of *p* < 0.05. *r* = Pearson correlation coefficients, MOE = modulus of elasticity, BS = bending strength, DS = density, SK = shrinkage, WA = water absorption, and VS = volume swelling.

**Table 4:** Average thermal conductivity values of MCBs obtained in this study.

Specimen Name	Thermal Conductivity (W/m∙K)
24C100S0	0.037
24C75S25	0.048
24C50S50	0.052
24C25S75	0.054
24C0S100	0.079

**Table 5:** Average sound absorption coefficient at each frequency for MCBs obtained in this study.

Specimen Name	Sound Absorption Coefficient (%)		
	250 Hz	500 Hz	1000 Hz
24C100S0	40	34	85
24C75S25	51	49	94
24C50S50	43	45	86
24C25S75	43	43	84
24C0S100	40	41	61

**Table 6:** Comparison of MBC properties in this study with commercial boards.

Properties		MBCs		Commercial Boards *					
		This Study	Previous Studies	Medium-Density Fiber Board	Softboard	Gypsum Board	Fiber Cement Board	Insulated Board	Acoustic Board
MOE (MPa)		0.17–7.88	0.14–97.00	2500–4000	80–150	1500–3000	6000–15,000	3000–7000	20–100
BS (MPa)		0.13–1.26	0.02–4.40	20–40	0.70–1.20	1.5–3.5	10–30	0.1–0.3	0.5–2.0
DS (kg/m^3^)		209.17–256.28	25–954	640–800	230–400	600–800	1300–1700	15–200	60–400
SK (%)		8.15–25.78	2.78–17	0.01–0.30	0.2–0.5	0.05–0.10	0.02–0.05	0.015–0.030	0.02–0.10
WA (%)		96.86–254.11	24.45–560	5–15	30–70	30–50	10–25	1–4	5–30
VS (%)		2.63–19.03	0.28–21	10–25	15–40	0.02–0.10	0.1–2.0	1.0–2.5	0.1–1.0
TC (W/m·K)		0.037–0.079	0.029–0.124	0.10–0.18	0.035–0.060	0.16–0.25	0.20–0.40	0.022–0.040	0.03–0.05
SAC (%)	250 Hz	40–51	16–20	10–20	15–30	5–10	10–20	30–60	50–80
	500 Hz	34–49	50–51	8–15	30–50	5–8	8–15	65–90	80–100
	1000 Hz	61–94	70–75	6–12	50–70	4–7	6–12	85–100	90–100

MOE = modulus of elasticity, BS = bending strength, DS = density, SK = shrinkage, WA = water absorption, VS = volume swelling, SAC = sound absorption coefficient. * Seddeq [80], Hsu et al. [84], Cai et al. [85], Ayrilmis et al. [86,87,88], Suchsland et al. [89], Sonderegger et al. [90], Nemli et al. [91], BS EN 622-4:2009 [92], Papadopoulos and Hill [93], Ganev et al. [94], Berardi et al. [95], Cramer et al. [96], Bénichou et al. [97], Karni et al. [98], Wakili et al. [99], Camino et al. [100], Feng et al. [101], Warnock [102], Ardanuy et al. [103], Mohr et al. [104], Akers. [105], Biot et al. [106], Jelle [107], Papadopoulos [108], Schiavoni et al. [109], and Asdrubali et al. [110].

## Data Availability

The original contributions presented in the study are included in the article, further inquiries can be directed to the corresponding author.
